# Learning to suppress a distractor is not affected by working memory load

**DOI:** 10.3758/s13423-019-01679-6

**Published:** 2019-12-03

**Authors:** Ya Gao, Jan Theeuwes

**Affiliations:** 1 Department of Experimental and Applied Psychology, Vrije Universiteit Amsterdam, Van der Boechorststraat 7, 1081 BT Amsterdam, The Netherlands; 2 Institute Brain and Behavior Amsterdam (iBBA), Amsterdam, The Netherlands

**Keywords:** Attentional capture, Visual search, Working memory, Statistical regularities

## Abstract

Where and what we attend to is not only determined by our current goals but also by what we have encountered in the past. Recent studies have shown that people learn to extract statistical regularities in the environment resulting in attentional suppression of high-probability distractor locations, effectively reducing capture by a distractor. Here, we asked whether this statistical learning is dependent on working memory resources. The additional singleton task in which one location was more likely to contain a distractor was combined with a concurrent visual working memory task (Experiment 1) and a spatial working memory task (Experiment 2). The result showed that learning to suppress this high-probability location was not at all affected by working memory load. We conclude that learning to suppress a location is an implicit and automatic process that does not rely on visual or spatial working memory capacity, nor on executive control resources. We speculate that extracting regularities from the environment likely relies on long-term memory processes.

**Electronic supplementary material:**

The online version of this article (10.3758/s13423-019-01679-6) contains supplementary material, which is available to authorized users.

In everyday life, we focus our attention to events that are relevant to us and ignore information that could distract us. For example, when biking along a busy road in Amsterdam, one needs to focus on other road users, such as cyclists and pedestrians, attend to traffic signs while ignoring the loud flashing billboards, or ignore the buzzer from your phone. The overload of input requires filtering and attenuation, allowing us to focus on relevant information and ignore distracting information. For many years, it was assumed that attentional selection was the result of the interplay between goals of the observer (top-down selection) and the physical salience of the visual environment (bottom-up selection; Theeuwes, 2010).

Recently, it was argued that, in many cases, objects are selected that are not part of the goal set of the observer, nor are they salient enough to capture attention automatically (Awh, Belopolsky, & Theeuwes, 2012; Failing & Theeuwes, 2018; Theeuwes, 2018, 2019). A third category of selection was suggested, called “selection history,” referring to the notion that previous attentional deployments can elicit lingering and enduring selection biases that are unrelated to the current goals or to the stimulus-driven saliency of objects (Theeuwes, 2018, 2019). It should be noted that the role of “selection history” on attentional selection was recognized before; yet, in many cases, it was considered to be a form of top-down selection (Navalpakkam & Itti, 2006; Wolfe, Butcher, Lee, & Hyle, 2003).

Recent studies provide compelling evidence regarding the role of selection history in biasing attentional selection. Wang and Theeuwes (2018a, 2018b) used the classic additional singleton task and showed that statistical regularities regarding the location of the distractor affected selection. In these studies, participants searched for a salient shape singleton (i.e., a diamond between circles or a circle between diamonds) while ignoring a colored distractor singleton. Unknown to the participant, the distractor singleton was presented systematically more often in one location than in all other locations. These studies collectively showed several important results: (1) When presented at the high-probability location, the color singleton distractor caused less attention capture than when presented at any of the regular locations; (2) when the target singleton was presented at this high-probability location, its selection was impoverished, as the time to select the target was longer when presented at this location than at all other locations; and (3) there was a spatial gradient of suppression from this high-probability location as the attentional capture effect scaled with the distance from this location. On the basis of these studies, it was concluded that through statistical learning, the location that is highly likely to contain a distractor becomes suppressed relatively to all other locations (Ferrante et al., 2018; see Gaspelin & Luck, 2018, for a review on distractor suppression; Wang & Theeuwes, 2018a, 2018b). Specifically, it was argued that this type of suppression is *proactive,* as this location is already suppressed before the search display was presented (Wang, van Driel, Ort, & Theeuwes, 2019).

All these studies show that statistical learning has a dramatic effect on attentional selection. Crucially, even though the effects on selection are dramatic, participants have little, if any, awareness regarding the regularities present in the display. For example, in Wang and Theeuwes (2018b), very few participants could report with certainty which location contained the distractor most often, even though the distractor was presented at that location 13 times more often than at all other locations. Similarly, Ferrante et al. (2018), who used a slightly different paradigm, indicated that participants were basically unable to explicitly report the statistical regularities they introduced in the display.

These findings have led to the notion that the statistical regularities present in the display bias attention in an implicit way (Feldmann-Wüstefeld & Schubö, 2013; Ferrante et al., 2018; Goschy, Bakos, Müller, & Zehetleitner, 2014; Wang & Theeuwes, 2018b). It is consistent with the claim that this type of selection is not the result of explicit top-down goals, but instead is learned unintentionally, automatically, without much, if any, explicit awareness (Theeuwes, 2018, 2019). If this is the case, then one expects that occupying the cognitive system with other secondary explicit tasks should not matter much, because these automatic implicit processes are assumed to take place regardless of the cognitive, explicit load that is required.

The present study investigated the role of cognitive load (cf. working memory load) in acquiring these selection biases. To that end, we employed the additional singleton paradigm, in which the salient distractor was presented much more in one location than in all other locations (see Wang & Theeuwes, 2018b). The task was performed under low-working-memory versus high-working-memory-load conditions. The predictions are straightforward: If learning to suppress the salient distractor is truly an automatic process, then we expect basically the same performance under low-working-memory versus high-working-memory conditions. If, however, learning to suppress the distractor requires some resource-dependent executive control process, one can expect that the distractor is not suppressed, at least not to the same extent it would be as under low or no-working-memory conditions.

Previous research has demonstrated that attentional capture is modulated by resource-dependent executive control processes, such as working memory. For example, physically salient but task-irrelevant stimuli capture more attention under higher working memory load (Burnham, Sabia, & Langan, 2014; Lavie & De Fockert, 2005) because under high working memory there is less executive control, which in turn reduces the ability to suppress task-irrelevant information. Given these findings, one expects that under high-load conditions, the suppression of the high-probability location is less optimal, as there is less attentional control. However, if this suppression is truly implicit, and not resolved by frontally mediated executive functions (Baddeley & Della Sala, 1996; D’esposito & Postle, 2015), then one expects that participants learn to suppress this location regardless of the working memory load.

## Experiment 1

### Method

#### Participants

Twenty-four participants (19 females, *M*_age_ = 20, *SEM* = 1.72 years) were tested for course credit or payment of 9 €.

#### Apparatus

Participants were tested in a dimly lit laboratory and held their chins on a fixed chin rest, 72 cm from the screen. The experiment was controlled by OpenSesame Version 3.2.5 (Mathôt, Schreij, & Theeuwes, 2012) and run on an HP Compaq Pro 6300 SFF computer with a 22-inch liquid crystal display (LCD) color monitor (1,680 × 1,050 pixel resolution, 120-Hz refresh rate).

#### Design

The visual search task was based on Wang and Theeuwes (2018b). Each trial started with a 500-ms white central cross (0.55° × 0.55°), followed by a search display consisting of a set of eight shapes (seven circles and one diamond, or vice versa) in either one of two colors (red—RGB: 220, 0, 0, luminance: 22 cd/m^2^; green—RGB: 0, 180, 0, luminance: 25 cd/m^2^). All eight shapes were presented on an imaginary circle with a radius of 4.5°, and each shape (about 2° diameter) contained a vertical or horizontal gray line (1.6°). Participants searched for the unique shape and responded to the line inside of it by pressing the *left* or *up* keys. A buzzer sounded when an error was made. Each block comprised 80 trials, with 24 trials (30%) in which all shapes in the search display were of the same color (no-distractor condition), 35 trials (43.75%) in which the distractor in the search display only presented at one specific location (high-probability locations), and 21 trials (26.25%) in which the distractor in the search display presented at other seven locations (low-probability location). For each memory load condition (high vs. low), a different location served as the high-probability location.

**Fig. 1:**
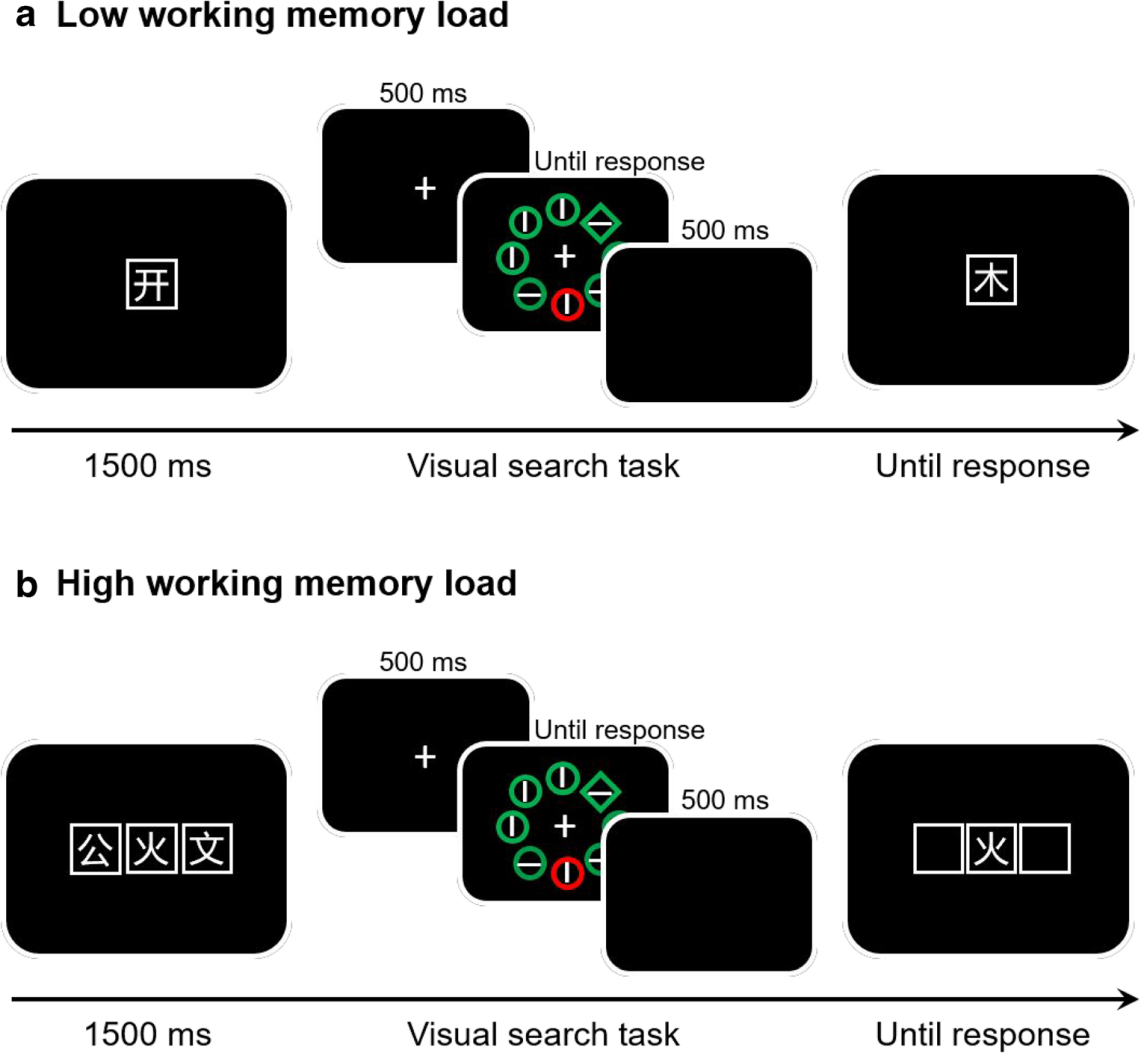
Experimental procedures in Experiment 1. **a** In the low working memory condition, participants needed to remember one Chinese character. After a 500-ms fixation display, a search display was presented. Participants were required to search for the different shape stimuli (target) and ignore the different color stimuli (distractor). The search display was present for 3 s or until response for the orientation of line inside of the target. After a 500-ms blank, the memory probe appeared at the center, and participants were prompted to determine whether the probe character was identical to the memory character. **b** In the high working memory condition, participants were required to remember three characters and their locations, and the probe character can be in any location. Participants needed to determine the character and its location

The working memory task used here to manipulate working memory load was similar to that used by Lavie and De Fockert (2005). Instead of digits, we used five Chinese characters as memory materials (i.e., “公”; “开”; “木”; “火”; “文”). These five characters all have four strokes and bilateral symmetry structure. In high working memory, load blocks (see Fig. 1b), we randomly chose three of them as a memory set and placed them randomly into three white squares (2.2° × 2.2°). Each character subtended 1.4° × 1.4°, and the whole memory set subtended 4.1° to the left and right of fixation. In the high-working-memory-load condition, participants had to remember the three characters and their locations; in the low load, only one character and its location. Following a visual search trial, participants determined whether or not a randomly placed probe letter was present in the memory array, and if so, whether it was positioned at the correct location (press “s” for same; press “d” for different). Five characters occurred equally often in the memory sets and served equally as the memory probe. Wrong responses or failure to respond within 5 s were followed by an error buzzer. The intertrial interval was between 500 and 750 ms at random.

#### Procedure

The experiment contained two sessions for each working memory load condition, and each session comprised four blocks of 80 trials. The session sequence was counterbalanced across participants. Before each session, participants completed 36 practice trials with the visual search task, only for the no-distractor condition. A feedback screen of mean accuracies and average response times (RTs) for the two tasks was presented after each block, and participants were asked to answer two questions after the whole experiment. They were asked to indicate if they had noticed any regularity regarding the location of the distractor, and irrespective of their answer had to indicate one location in each session on the search display where they thought that the distractor appeared more often.

### Results

Trials in which the RTs were larger than 2.5 standard deviations from the average response time per working memory load condition per participant or less than 200 ms were excluded from the RT analyses. We quantified the Bayes factor (BF) using Bayesian hypothesis testing in JASP (JASP Team, 2018) to better evaluate the strength of the evidence for the alternative hypothesis (H_1_) over the null hypothesis (H_0_) whenever a comparison using traditional null hypothesis testing was nonsignificant.

#### Memory task

Participants made significantly more errors in the high-working-memory-load condition (*M* = 14.03%) than in the low-load condition (*M* = 6.79%), *t*(23) = 4.985, *p* < .001.

#### Attentional capture effect

**Fig. 2:**
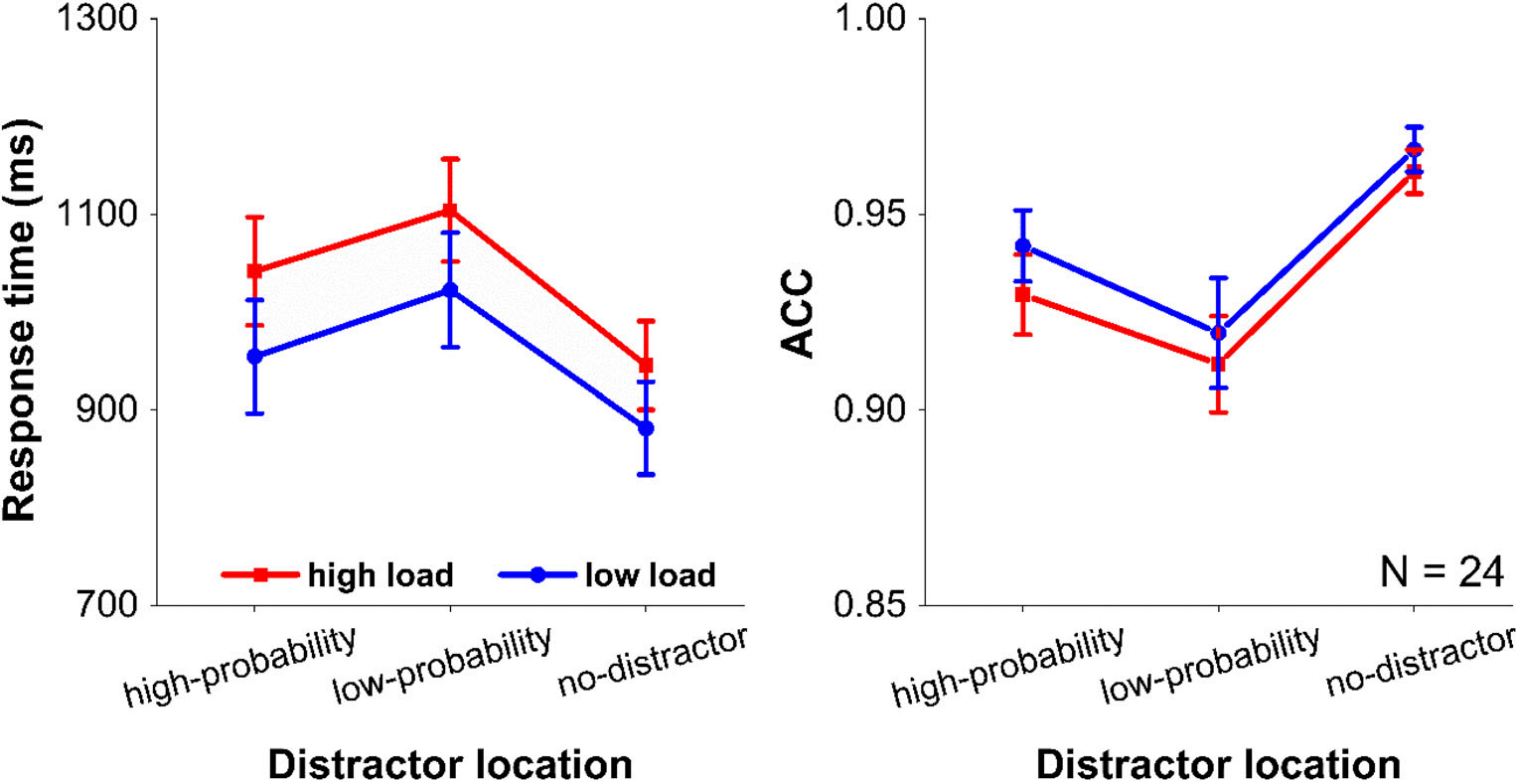
Results of Experiment 1. The mean response times (left panel) and the mean accuracies (ACC; right panel) between the different distractor conditions under the low and the high working memory load conditions. Error bars denote ±1 standard error of the mean

Mean RTs and mean accuracies are presented in Fig. 2. We performed a 2 × 3 repeated-measures ANOVA on mean RTs and mean accuracies, with working memory load (high vs. low) and the distractor condition (high-probability locations, low-probability locations, and no distractor) as two within subject factors. Only trials with a correct response on both the visual search task and the memory task were used in the RT analyses.

There was a main effect of working memory load. *F*(1, 23) = 5.235, *p* = .032, η_p_^2^ = 0.185, with faster RTs in the low-load than in the high-load condition. The main effect of distractor condition was also significant, *F*(2, 46) = 65.410, *p* < .001, η_p_^2^ = 0.74. Further paired *t*-test analyses under both working memory load conditions showed the same effects: RTs were significantly slower when distractors were presented at high-probability locations (*p*s < .001), and low-probability locations (*p*s < .001), relative to the no-distractor condition. There was also a reliable difference between high and low-probability locations, *t*(23) = 3.962, *p* = .001, for the high load; *t*(23) = 6.694, *p* < .001, for low load). These results indicate that attentional capture was modulated by the location of the distractor. Critically, the interaction between these two factors was unreliable, *F*(2, 46) = 0.798, *p* = .456, η_p_^2^ = 0.034, BF_01_ = 7.7. The Bayes factor indicates that there is moderate to strong evidence for the H_0_. This indicates that the attenuation of capture for distractors presented at the high-probability location was equally strong for the high and the low-memory-load condition. In other words, regardless of memory load, participants were equally effective at learning to suppress the location that was more likely to contain a distractor. Accuracies basically followed RTs (see Fig. 2).

Identical to Wang and Theeuwes (2018a), we found that when the target singleton happened to be presented at the high-probability location, its selection was impoverished, as the time to select the target was longer when presented at this location than at all other locations (Supplemental Information 1). In addition, we also found a spatial gradient of suppression from this high-probability location as the attentional capture effect scaled with the distance from this location (Supplemental Information 1). Finally, our findings are not the result of intertrial priming (Supplemental Information 1).

#### Awareness assessment

When asked whether they noticed anything regarding the regularities of the location of the distractor, one participant indicated noticing that some locations contained the distractor more often than other locations. When forced to indicate which locations, this participant and another participant indicated correctly both high-probability locations used in the high versus low memory load conditions. We excluded these two data and conducted the RT analysis again, and the results did not change (see Supplemental Information 1). Overall, this analysis suggests that there is little evidence (if any) that participants were aware of the regularities.

### Discussion

Whether or not participants have a high-working-memory load or a low-memory load, they equally effectively learn to suppress the location that is most likely to contain the distractor. If the target happens to be presented at that location, it is also suppressed. The suppression results in less attentional capture that scales with the distance from the high-probability location. The current findings for both the high-load and low-load conditions are basically identical to the results to Wang and Theeuwes (2018a, 2018b). It should be noted that our manipulation of working memory load was effective, as participants had much slower RTs (about 77.6 ms overall) in the high-load condition than in the low-load condition. So even though there was clear working memory load, learning was equally effective. It indicates that the reduction in capture due to statistical learning is not dependent on executive control processes that are assumed to be dependent on working memory. This result is consistent with the notion that this type of learning is implicit, occurring without much, if any, awareness.

**Fig. 3:**
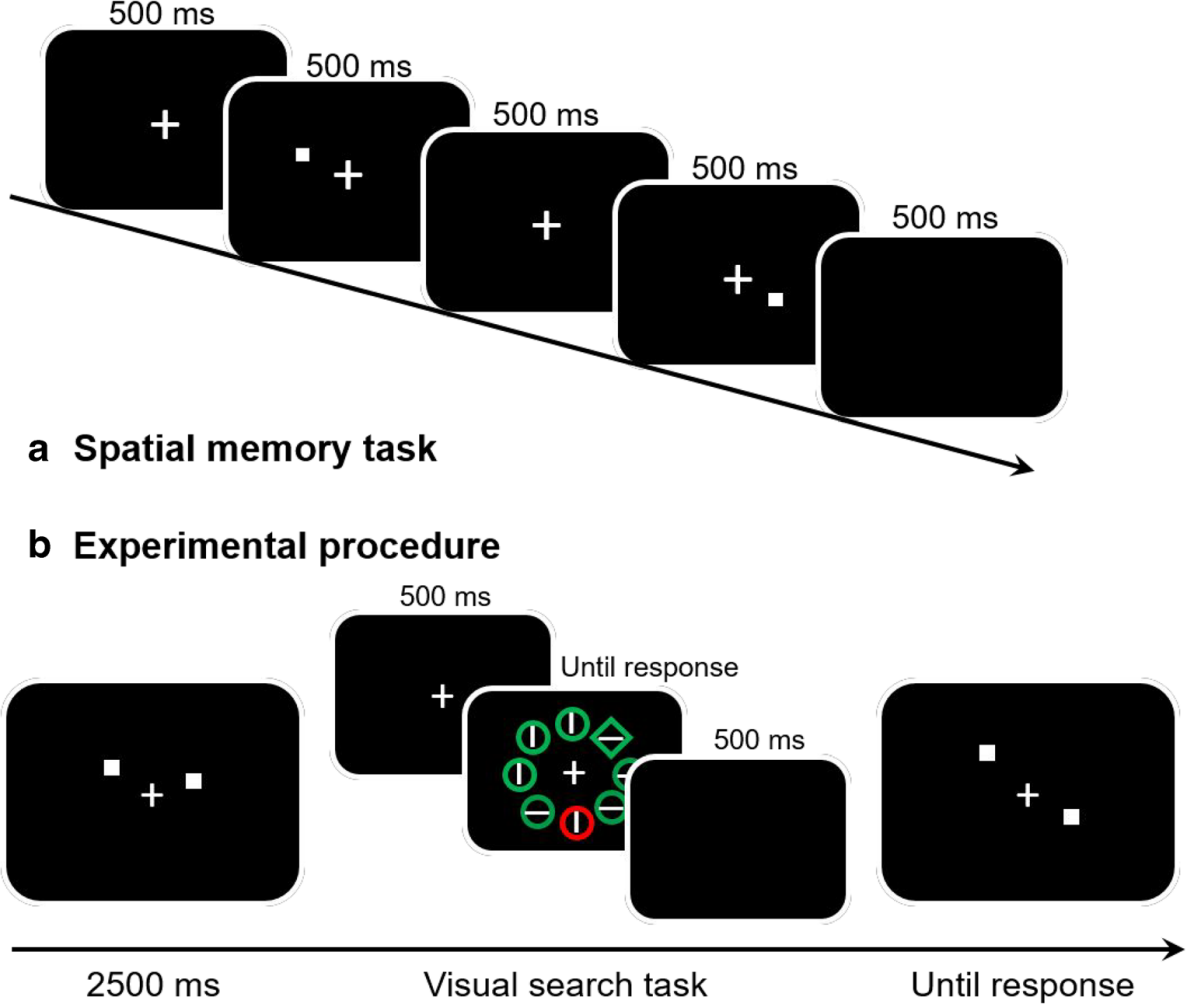
Experimental procedures in Experiment 2. a The spatial working memory condition. Participants were asked to remember two locations that were indicated by two sequentially presented white squares. **b** The procedures in Experiment 2 was the same as in Experiment 1. In the spatial working memory condition, participants were required to remember two locations at the beginning of each trial and performed a location change detection task after the visual search task; in the no spatial working memory condition, participants were instructed to ignore the white squares and only do the visual search task

Even though the results of Experiment 1 are quite convincing, one may argue that the type of working memory load that we introduced may not have affected statistical learning during visual search because it did not involve visual *spatial* memory. Indeed, even though the memory load in Experiment 1 was clearly visual (verbal recording of Chinese characters by non-Chinese participants is unlikely), the spatial component was somewhat limited. To fully engage visual *spatial* working memory, we employed as a secondary task—a spatial working memory task as was used by Woodman and Luck (2004), where participants have to remember the exact location of two sequentially presented probes (see Fig. 3). The sequential presentation prevents participants from trying to store some configuration representation image in their visual working memory. Woodman and Luck (2004) showed that with this secondary task, visual search was very much impaired, while this was not the case when participants only had to keep visual object representations in working memory.

The predictions are straightforward: If learning to suppress a particular location is not affected by whether or not spatial working memory is occupied, then we expect equally effective suppression regardless of whether participants are keeping two locations in spatial working memory. Alternatively, it is possible that occupying spatial working memory hampers statistical learning, resulting in less suppression of the high-probability location when spatial working memory is occupied by a secondary task than when it is not occupied.

## Experiment 2

Experiment 2 was identical to Experiment 1, except that we used a truly spatial working memory task (the same task as Woodman & Luck, 2004). We examined whether spatial working memory was involved with statistical learning process.

### Method

Twenty-four new healthy adults (15 females, *M*_age_ = 20.95, *SEM* = 2.36 years) participated in Experiment 2. The experiment was identical to Experiment 1, except that a spatial working memory task was used, identical Woodman and Luck (2004).

As is shown in Fig. 3, after a 500 ms fixation, participants were asked to remember two locations that were indicated by two sequentially presented white squares (0.3° × 0.3°). Each square was presented for 500 ms, and the two squares were separated by a 500-ms interval. Squares could appear at 36 possible locations, which were divided into four quadrants. In each quadrant, the square was positioned randomly at the intersections of an imaginary 4 × 4 grid (4° × 4°). In each trial, the two squares were at least 1.4° away from the fixation cross (center-to-center distance). Participants had to remember these two locations in the spatial working memory condition and ignore these two locations in the no-spatial-working-memory condition. After each trial, the memory test display was presented, with two squares shown simultaneously. In spatial working memory, participants judged whether the test squares’ locations had changed. They responded by pressing the “s” key for constant locations and the “d” key for changed locations (only one location changed). In the no-spatial-working-memory condition, participants were instructed to press “space” to continue.

### Results

As in Experiment 1, trials in which the RTs were larger than 2.5 standard deviations from the mean RTs per memory condition per participant and RTs less than 200 ms were excluded from the RT analyses.

#### Memory task

Mean accuracy in spatial working memory was 78.36%, significantly above chance, *t*(23) = 28.106, *p* < .001 (one-sample *t* test compared with 50%).

#### Attentional capture effect

**Fig. 4:**
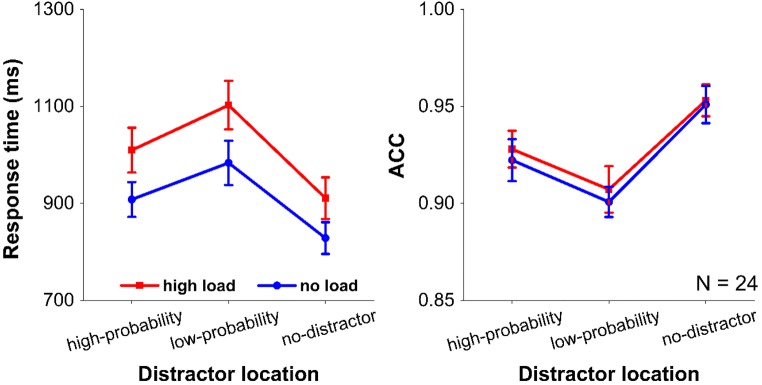
Results of Experiment 2. The mean response times (left panel) and the mean accuracies (ACC; right panel) between the different distractor conditions under no and spatial working memory conditions. Error bars denote ±1 standard error of the mean

The main effect of load was significant, *F*(1, 23) = 6.06, *p* = .022, η_p_^2^ = 0.209, with slower responding when participants kept two locations in spatial working memory than when they had nothing in working memory. The main effect of distractor condition was also significant, *F*(2, 46) = 110.402, *p* < .001, η_p_^2^ = 0.828. Further paired *t*-test analyses under both memory conditions showed that RTs were significantly slower when distractors were presented at high-probability locations (*p*s < .001), and low-probability locations (*p*s < .001), relative to the no-distractor condition. There was also a significant difference between high and low probability locations (*p*s < .001). Again, no significant interaction was found between the two factors, *F*(2, 46) = 1.747, *p* = .186, η_p_^2^ = 0.071, BF_01_ = 7.5. The results indicate that it does not matter whether participants hold items in spatial working memory or whether spatial working memory is not taxed at all: In both conditions, there was less capture by the distractor presented at the high-probability location relative to the low-probability location. Accuracies basically followed RT (see Fig. 4).

Again, we also found that when the target was presented at the high-probability location, RTs were slowed, and we found a gradient of suppression and showed that it is not due to intertrial priming (see Supplemental Information 2).

#### Awareness assessment

None of the participants noticed anything. When forced to indicate locations, four participants indicated correctly both high-probability locations used in the two conditions. When we excluded these four participants, the results remained the same, indicating that awareness of knowing the distractor regularity did not alter the results (see Supplemental Information 2).

### Discussion

The results of Experiment 2 are basically identical to Experiment 1. Even though we have now introduced a secondary task that heavily taxes spatial working memory, participants were equally effective in learning to suppress the high-probability location when spatial working memory was full versus when spatial working memory was not occupied at all. This result is remarkable, given the strong link between spatial attention and visuospatial working memory (Awh & Jonides, 2001; Postle, 2006; Theeuwes, Belopolsky, & Olivers, 2009; Van der Stigchel, Merten, Meeter, & Theeuwes, 2007).

## General discussion

The current findings are clear: Whether or not visual object representations (Experiment 1) or specific locations (Experiment 2) are stored in working memory, participants are equally effective in learning to suppress the location most likely to contain a distractor. Even though this conclusion of equally effective learning, regardless of whether visual working memory is taxed or not is based on the absence of an interaction between load and distractor location, it should be that in both experiments the visual working memory (VWM) load manipulation was highly successful, as in both experiments there were clear main effects of VWM load. Also, the large Bayes factors regarding this interaction indicate reasonable evidence for this null effect.

The current results are important, as they indicate that this type of learning does not depend on executive control processes that are assumed to rely on working memory (Baddeley & Della Sala, 1996). It further strengthens the idea that this type of suppression resulting from learning does not rely on effortful and volitional top-down control processes (Theeuwes, 2018, 2019), as it is generally agreed that working memory load interferes with top-down attentional control (Gazzaley & Nobre, 2012). It confirms the notion that the type of learning is implicit and automatic, not relying on executive processing resources. Consistent with the notion of implicit learning, in the current study, participants were not aware of the regularities that were introduced, a result which was reported before (Ferrante et al., 2018; Wang & Theeuwes, 2018a, 2018b, 2018c).

The absence of an effect of spatial working memory load on this type of learning (our Experiment 2) is even more noteworthy. It is well known that there is a strong link between attention and spatial working memory (Awh & Jonides, 2001; Cowan et al., 2005; Postle, 2006; Theeuwes et al., 2009). For example, Awh, Jonides, and Reuter-Lorenz (1998) showed that storing and holding a location in working memory is accomplished by shifting spatial attention to that location in space (see also Theeuwes, Kramer, & Irwin, 2011). Also, it was shown that when attention to memorized locations was interrupted, the ability to remember these locations was impaired. Woodman and Luck (2004), who employed the same spatial working memory task as we have used here, showed that this secondary task interfered with the efficiency of visual search. Brain imaging studies of working memory confirm the notion that rehearsal of spatial information modulates early sensory areas (Awh & Jonides, 2001; Munneke, Heslenfeld, & Theeuwes, 2010). So even though the link between spatial working memory and spatial attention is undisputed, our study shows that learning to suppress a location in space is not affected by whether or not spatial working memory is occupied.

The current task is in some way related to “contextual cueing,” which has demonstrated that search for a target is facilitated when it appears in a visual layout that was previously searched relative to visual layouts that were never seen before (Chun & Jiang, 1998; Chun & Phelps, 1999; Jiang & Chun, 2001). Manginelli, Langer, Klose, and Pollmann (2013) investigated the effect of VWM load on contextual cueing. In their study, participants had to search for *T*s among *L*s, and in 50% of trials, particular displays were repeated. This search task was combined with either a visuospatial or nonspatial working memory task during an initial learning phase and during a test phase. Consistent with the current findings, nonspatial working memory load had no effect on performance, independent of whether it was presented in the learning or test phase. However, unlike the present findings, visuospatial load had a negative effect during the test phase, reducing the contextual cueing effect. During learning, the visuospatial working memory load had no effect. Manginelli et al. (2013) concluded that visuospatial working memory is needed for the expression of previously learned spatial contexts (but see Won & Jiang, 2015, for a different opinion). This conclusion is inconsistent with the current findings, which show that the expression of learning is found even under high VWM load conditions. Note, however, that the current task is very much unlike a classic contextual cueing task. One evident difference is that in our task, the salient singleton distractor stands out from the background, calling attention to the location in an automatic bottom-up way (Theeuwes, 1992), while in typical contextual cueing tasks (e.g., searching *T*s among *L*s), no particular element stands out from the background, and search is generally serial in nature.

As working memory has no immediate impact on learning, one may speculate how learning is accomplished. One option that comes to mind is to assume that long-term memory plays a role. For example, in contextual cueing, the role of long-term memory has been implicated. In contextual cueing, participants are able to detect targets appearing in repeated configurations more quickly than in novel configurations (e.g., Chun & Jiang, 1998). Specifically, it was suggested that the neural circuitries of the hippocampus and medial temporal lobe could be candidates for encoding contextual information in the brain (Turk-Browne, Scholl, Chun, & Johnson, 2009; Vickery, Sussman, & Jiang, 2010), explaining why there is little awareness of which configurations are repeated in contextual cueing (Chun & Phelps, 1999).

In sum, the current study shows that learning to suppress a location that is likely to contain a distractor is an implicit, automatic process that does rely on visual or spatial working memory capacity, nor on executive control resources. It is not an intentional process, and occurs mostly outside of awareness. Encoding of locations that may contain distracting information most likely relies on long-term memory processes, specifically in the hippocampal areas of the medial temporal lobe.

### Author note

This research was supported by a China Scholarship Council (CSC) scholarship to Y.G. The data and analysis script will be offered upon request.

## Electronic supplementary material

ESM 1 (DOCX 44 kb)

ESM 2 (DOCX 43 kb)
