# Supplementary material for: Learning to suppress a distractor is not affected by working memory load
Source: Psychon Bull Rev. 2019 Dec 3;27(1):96–104. doi: 10.3758/s13423-019-01679-6 (PMC7000502; doi:10.3758/s13423-019-01679-6)
Supplement: Supplementary file 1 — (DOCX 44 kb) [file 13423_2019_1679_MOESM1_ESM.docx]

**Exp.1**

**Additional results**

**Intertrial location-based priming analysis**

To rule out the possibility that this suppression effect for distractors presented at the high probability versus the low probability location was due to intertrial location-based suppression (priming) effect, we excluded all trials in which the location of the distractor was repeated from one trial to the next. Then we compared this part of results with the original data that contained the repeated trials, and performed a 2×2×2 repeated measures ANOVAs on mean RTs, with working memory load (high vs. low), distractor condition (high-probability locations vs. low-probability locations) and group (with repeated trials vs. without repeated trials) as three within subject factors. We only found a main effect of distractor condition (F(1,23) = 37.335, p < 0.001, η2p = 0.619), and a main effect of working memory load (F(1,23) = 4.859, p = 0.038, η2p = 0.174). There was no effect of group, nor any interaction with the other variables. This shows that the suppression effect is unaffected by intertrial priming.

**Target at the high probability distractor location**

As shown in Fig. S1, we performed a 2×2 repeated measures ANOVAs on mean RTs and mean accuracies in no distractor condition, with working memory load (high vs. low) and target location (high-probability distractor location vs. low-probability distractor location) as two within subject factors. For RTs, the main effect of working memory load showed a marginal significance, F(1,23) = 3.722, p = 0.066, η2p = 0.139. Importantly, the main effect of target location was significant, F(1,23) = 4.824, p = 0.038, η2p = 0.173, indicating that even when the distractor was absent, participants’ responses were still influenced by the high probability distractor location, they respond slower when target appeared in the high probability distractor location relative to low probability locations. No interaction between these two factors was found (F(1,23) = 0.227, p = 0.638, η2p = 0.01). We also observed a main effect of target location on mean accuracies, F(1,23) = 4.201, p = 0.052, η2p = 0.154. Besides, no other effects were statistically significant, Fs < 0.392, ps > 0.537.

Fig. S1 The mean response times (left panel) and the mean accuracies (right panel) when target at different distractor locations under low and high working memory load conditions. Error bars denote ±1 the standard error of the mean.

**The spatial gradient of the suppression effect**

Wang and Theeuwes (2018) reported a spatial gradient of the suppression effect. The distractor was most suppressed when it was presented at the high probability location, and this suppression effect had a spatial distribution such that suppression became less the further away from the high probability location. In the current experiment, we found the same effect under both high and low working memory load conditions. Mean RTs and mean accuracies for each distance are presented in Fig. S2. We conducted a 2×5 repeated measures ANOVAs for the mean RTs and mean accuracies, with working memory load (high vs. low) and distance (dist-0, dist-1, dist-2, dist-3, and dist-4) as two within subject factors. For results on mean RTs, the main effect of working memory load was significant, F(1,23) = 4.469, p = 0.046, η2p = 0.163, as a result of faster responses in low working memory load condition. The main effect of distance was significant, F(4,92) = 7.209, p < 0.001, η2p = 0.239. Crucially however, no interaction was found between these two factors, F(4,92) = 0.389, p = 0.816, η2p = 0.017. There were no significant effects on mean accuracies, Fs < 1.554, ps > 0.193. To describe the nature of this trend on RTs, a linear function was fitted for data from dist-0, the high probability location to dist-4. The slope (19.373 ms per point of distance) under high working memory load was significantly larger than zero, t(23) = 2.307, p = 0.03. And the slope (15.384 ms per point of distance) under low working memory load was also significantly larger than zero, t(23) = 2.67, p = 0.014. There was no difference between these two conditions, t(23) = 0.371, p = 0.714.

Fig. S2 The spatial distribution of attentional capture effect by the means of response times (RTs; left panel) and accuracies (right panel) under high and low working memory load conditions. Here, dist-0 refers to the high probability location, and dist-4 refers to the location on the opposite side of the imaginary circle. Error bars denote ±1 the standard error of the mean.

**Awareness assessment**

When excluding two participants’ data who correctly identified the high probability distractor location, the main effect of memory load (F(1,21) = 6.347, p = 0.02, η2p = 0.232) was still significant, with faster RTs in low load than in high load condition. The main effect of distractor condition was also significant, F(2,42) = 62.462, p < 0.001, η2p = 0.748, indicating that attentional capture was modulated by the location of the distractor. Critically, the interaction between these two factors was unreliable (F(2,42) = 0.936, p = 0.4, η2p = 0.043, *BF*_01_ = 7.1), which indicates that the attenuation of capture for distractors presented at the high probability location was equally strong for the high and the low memory load condition.
