# Supplementary material for: Learning to suppress a distractor is not affected by working memory load
Source: Psychon Bull Rev. 2019 Dec 3;27(1):96–104. doi: 10.3758/s13423-019-01679-6 (PMC7000502; doi:10.3758/s13423-019-01679-6)
Supplement: Supplementary file 2 — (DOCX 43 kb) [file 13423_2019_1679_MOESM2_ESM.docx]

**Exp.2**

**Additional results**

**Intertrial location-based priming analysis**

In order to rule out intertrial location-based priming, we excluded all trials in which the location of the distractor repeated from one trial to the next, and compared with the original data that contained the repeated trials. A 2×2×2 repeated measures ANOVAs on mean RTs was performed, with spatial working memory load (present vs. absent), distractor condition (high-probability locations vs. low-probability locations) and group (with repeated trials vs. without repeated trials) as three within subject factors. We observed a main effect of group (F(1,23) = 7.104, p = 0.014, η2p = 0.236) and a marginal significant interaction between group and distractor condition (F(1,23) = 3.916, p = 0.06, η2p = 0.145), which indicates a contribution to the effect of intertrial priming. Yet, regardless there was still a reliable main effect of distractor condition (F(1,23) = 55.538, p < 0.001, η2p = 0.707) and working memory load (F(1,23) = 6.115, p = 0.021, η2p = 0.21), which indicates that statistical learning occurs above and beyond intertrial priming.

**Target at the high probability distractor location**

As shown in Fig. S3, we performed a 2×2 repeated measures ANOVAs on mean RTs and mean accuracies in no distractor condition, with working memory load (high vs. no) and target location (high-probability distractor location vs. low-probability distractor location) as two within subject factors. For RTs, the main effect of working memory load was significant, F(1,23) = 5.370, p = 0.03, η2p = 0.189. The main effect of target location was significant, F(1,23) = 17.357, p < 0.001, η2p = 0.43, indicating slower responses when target appeared in the high probability distractor location relative to low probability distractor location. No interaction between these two factors was found (F(1,23) = 0.441, p = 0.513, η2p = 0.019). There was no effect no accuracies, Fs < 0.655, ps > 0.427.

Fig. S3 The mean response times (left panel) and the mean accuracies (right panel) when target at different distractor locations under no and high spatial working memory conditions. Error bars denote ±1 the standard error of the mean.

**The spatial gradient of the suppression effect**

We analyzed the spatial distribution of the suppression effect brought by the high probability distractor location. Mean RTs and mean accuracies for each distance away from the high probability location are presented in Fig. S4. We conducted a 2×5 repeated measures ANOVAs for the mean RTs and mean accuracies, with working memory load (high vs. no) and distance (dist-0, dist-1, dist-2, dist-3, and dist-4) as two within subject factors. For results on mean RTs, the main effect of working memory load was significant, F(1,23) = 6.522, p = 0.018, η2p = 0.221, as a result of faster responses in no working memory load condition. The main effect of distance was significant, F(4,92) = 12.673, p < 0.001, η2p = 0.355. Crucially however, no interaction was found between these two factors, F(4,92) = 0.311, p = 0.87, η2p = 0.013. The main effect of distance on mean accuracies also showed a significant effect, F(4,92) = 5.968, p < 0.001, η2p = 0.206; there were no other significant effects, Fs < 1.247, ps > 0.297. To describe the nature of this trend on RTs, a linear function was fitted for data from dist-0, the high probability location to dist-4. The slope (30.24 ms per point of distance) under high working memory load was significantly larger than zero, t(23) = 3.236, p = 0.004. And the slope (26.02 ms per point of distance) under no load condition was also significantly larger than zero, t(23) = 3.776, p = 0.001. There was no difference between these two conditions, t(23) = 0.339, p = 0.737.

Fig. S4 The spatial distribution of attentional capture effect by the means of response times (RTs; left panel) and accuracies (right panel) under high and no working memory load conditions. Here, dist-0 refers to the high probability location, and dist-4 refers to the location on the opposite side of the imaginary circle. Error bars denote ±1 the standard error of the mean.

**Awareness assessment**

We excluded four participants’ data who correctly identified the high probability distractor location at the end of the experiment and conducted the RT analysis again. The results showed that the main effect of memory load was significant, F(1,19) = 4.21, p = 0.054, η2p = 0.181, *BF*_10_ > 100, with slower responding when participants kept two locations in spatial working memory than when they had nothing in working memory. The main effect of distractor condition was also significant, F(2,38) = 107.539, p < 0.001, η2p = 0.85. Again, no significant interaction was found between two factors (F(2,38) = 1.711, p = 0.194, η2p = 0.083, *BF*_01_ = 6.1).
